# Nitrogen balance and yolk corticosterone levels of laying hens fed low-protein diets from 33 to 64 weeks of age

**DOI:** 10.5713/ab.250814

**Published:** 2025-12-18

**Authors:** Sunhoo Moon, Yun-Ji Heo, Jina Park, Da-Hye Kim, Kyung-Woo Lee

**Affiliations:** 1 Department of Animal Science and Technology, College of Life Sciences, Konkuk University, Seoul, Korea

**Keywords:** Corticosterone, Egg Production, Laying Hen, Low Protein, Nitrogen Excretion

## Abstract

**Objective:**

The study aimed to investigate the effects of varying dietary crude protein levels on laying performance, nitrogen balance, odor emission, and yolk corticosterone in laying hens from 33 to 64 weeks.

**Methods:**

Two hundred and forty laying hens (Hy-Line Brown) were randomly assigned to one of four varying protein diets from 18.5% to 12.5% at 33–45 weeks, from 18.0% to 12.0% at 46 to 55 weeks, and from 17.0% to 11.0% at 56 to 64 weeks.

**Results:**

Laying performance (i.e., egg weight, egg production, and egg mass, p<0.05) was linearly declined with decreasing dietary crude protein levels. Eggshell thickness decreased (quadratic effect, p = 0.033) at 44 weeks of age, while increased (linear effect, p<0.001) at 52 weeks of age as dietary protein levels lowered. Decreasing dietary protein levels linearly increased (p<0.05) Haugh unit at 52 and 64 weeks. Apparent digestibility of dry matter and crude protein increased at 45 and 55 weeks of age as dietary protein levels decreased. Lowering dietary protein levels increased the concentration of high-density lipoprotein cholesterol (linear effect, p = 0.012) but decreased (linear effect, p = 0.021) uric acid levels in serum samples at 64 weeks of age. Nitrogen excretion linearly declined as dietary protein levels decreased at all ages. Among the odors analyzed, fecal volatile fatty acids increased at 55 weeks of age with decreasing dietary protein levels. Finally, yolk corticosterone was not altered by dietary protein levels during the laying cycle.

**Conclusion:**

It is concluded that decreasing dietary protein levels fortified with limiting amino acids can be applied to mitigate nitrogen excretion without affecting nutrition-mediated stress in laying hens.

## INTRODUCTION

Excess dietary crude protein (CP) constitutes a major source of nitrogen excretion leading to environmental pollution in poultry industry [1,2]. Thus, low-protein diets fortified with limiting amino acids have been commonly employed as a nutritional strategy to mitigate nitrogen excretion [3]. However, excessive reductions in dietary protein levels could compromise laying performance and egg qualities [4–6], gut health (i.e., microbiota and intestinal morphology) [7,8], and immune responses [9] in laying hens. Indeed, Torki et al [10] reported that decreasing protein levels from 16.5% to 10.5% in the diets of laying hens from 52 to 60 weeks decreased egg production and egg mass, but increased serum triglyceride levels. Moreover, Bunchasak et al [11] reported that lowering dietary protein from 18% to 14% decreased protein conversion ratio and serum globulin including beta and gamma-globulin in laying hens. On the other hand, marginal reduction of protein levels by 1.4 percentage point in the diets of laying hens did not affect laying performance, egg qualities, and gut health indicators including concentrations of short-chain fatty acids in cecal digesta, mRNA expression of genes associated with gut barrier proteins (e.g., claudin-1, zonula occludens-1, and mucin-2 genes) in laying hens [12].

It is well documented that low CP diets play an important role in promoting sustainable poultry production by decreasing emissions of nitrogen, ammonia, nitrous oxide, and potentially methane and carbon dioxide from poultry feces [13–15]. In previous studies, low-protein diets lowered nitrogen excretion [16] and odor emission [14] in laying hens.

It is also well documented that nutrient deficiencies could alter physiological responses via disruption in gut health [17], antioxidant systems [18,19], or immune balance [20,21]. Recently, Park et al [22] reported that decreasing dietary phosphorus levels induced stress responses manifested by elevated concentrations of yolk corticosterone in laying hens. On the other hand, Heo et al [23] reported that low protein levels reduced yolk corticosterone in laying hens at 22 weeks of age, but the low protein-mediated reduction in yolk corticosterone was not seen at 26 and 32 weeks of age. These studies [22,23] suggest that yolk corticosterone levels, an indicator of stress responses in laying hens, could be influenced by different nutrient composition in early phases of laying production. It is however not known whether lowering protein levels in the diets of laying hens could affect stress response in mid- or late-stages of laying production. Thus, the present study was designed to test the effect of lowering protein levels on nitrogen balance, odor emission and yolk corticosterone in laying hens from 33 to 64 weeks of age. It is anticipated that the information obtained would be useful in assessing the long-term feeding of low-protein diets and unraveling nutrition-mediated alterations of stress response in laying hens.

## MATERIALS AND METHODS

### Experimental design, diets and birds

A total of 240 Hy-Line Brown laying hens aged 33 weeks of age were randomly subjected into one of four experimental diets with varying CP levels during three feeding phases (phase I from 33 to 45 weeks, phase II from 46 to 55 weeks, and phase III from 56 to 64 weeks). Four experimental diets were formulated to contain the graded CP levels at 2% intervals (Supplement 1) from 18.5% to 12.5% (phase I), from 18.0% to 12.0% (phase II), and from 17.0% to 11.0% (phase III). All experimental diets, in a powdered form, were ensured to have equal levels of metabolizable energy, limiting amino acids (lysine, methionine, threonine, and valine) and calcium. The hens were housed in three-tiered cages (45×45×45 cm per cage) equipped with a plastic trough feeder and nipple waterer. Each cage housed two hens, and three adjacent cages (six hens per replicate) were considered a replicate. Feed and water were provided *ad libitum* throughout the experiment. The experimental lighting schedule consisted of 16 h of light and 8 h of darkness (16L:8D).

Body weights were individually recorded at the beginning and end of the experiment and feed intake per replicate was measured at four-week intervals. Eggs laid were daily collected, weighed per replicate and used to calculate egg weight, egg production, and egg mass. The percentage of soft and broken eggs per replicate was calculated as total number of soft and broken eggs divided by total number of eggs multiplied by 100.

### Sample collection

At 64 weeks, one bird per replicate was randomly selected and euthanized with excess carbon dioxide. Blood was collected by heart puncture and serum was obtained by centrifugation (200×g for 15 min) and stored at −20°C until further analysis. Immediately after blood sampling, internal organs including abdominal fat, liver, spleen, bursa of Fabricius, ovary, oviduct, large yellow follicles, small intestine (duodenum, jejunum, ileum) and right leg were excised. A 1-cm-long mid-ileal segment was fixed in 10% neutral-buffered formalin for histological evaluation. Tibiae from the right leg were prepared by removing attached muscles and cartilage.

On the last three consecutive days at 36, 40, 44, 48, 52, 56, 60, and 64 weeks, 6 intact eggs per replicate were collected to quantify yolk corticosterone and eggs collected at 44, 56, and 64 weeks were used to measure egg qualities.

### Egg quality determination

Eggs collected at 44, 52, and 64 weeks were subjected to egg quality analysis (i.e., eggshell thickness, eggshell strength, Haugh unit, and yolk color score) using a digital egg tester (DET6000; Nabel). Eggshell color was measured by a shell color reflectometer (TSS QCR; Technical Services and Supplies).

### Organ weights, ileal morphology, and tibia characteristics

At 64 weeks, internal organs (i.e., abdominal fat, liver, kidney, spleen, duodenum, jejunum, ileum, bursa of Fabricius, ovary, and oviduct) collected were weighed and relatively expressed per body weight. Large yellow follicles over 0.5 cm per bird were counted and recorded. Villus height and crypt depth were measured in seven well-oriented villi at 40× magnification using a digital microscope (BX 43; Olympus), and images were captured with a digital camera (eXcope T500; Olympus). The width and length of the tibia were measured using a vernier caliper. Tibia breaking strength was measured using an Instron (Instron Universal Testing Machine Mo. 3342; Instron) with a 50-kg load range at a crossed speed of 50 mm/min with tibia supported on a 3.35 cm span.

### Nutrients digestibility and nitrogen balance

At 45, 55, and 64 weeks, nutrient digestibility and nitrogen balance were determined using the total fecal collection method. Each treatment consisted of 7 replicates with 2 birds per replicate. Birds were provided with free access to feed and water throughout the metabolism trial. Feed intake was recorded, and fecal samples were quantitatively collected twice daily for three consecutive days. Excreta from each replicate were pooled and stored at −20°C until further processing. Samples were subsequently dried in a forced-air oven at 65°C for 72 h and ground for chemical analyses. Feed and excreta samples were analyzed for [24] dry matter (method 930.15), CP (method 990.03), ether extract (method 920.39), and crude ash (method 942.05). Nitrogen balance including intake, excretion, and retention (difference between the intake and the excretion) was calculated according to the method described [22,23].

### Determination of odors in fecal samples

At 45, 55, and 64 weeks, odor emission was measured using the modified flux chamber method [25]. In short, approximately 150 g of freshly voided excreta were placed in a 6 L plastic container. To equilibrate odorant concentrations, nitrogen gas (99.99%) was introduced at a rate of 1 L/min for 10 min. Odor emissions were then analyzed with a Gastec detector (GV-100S; Gastec) equipped with detector tubes for CO_2_, NH_3_, H_2_S, and trimethylamine. In addition, the concentrations of total volatile fatty acids in fresh excreta samples were assayed using gas chromatography (6890 Series GC System; Hewlett Packard) per the method described elsewhere [26].

### Serum biochemical parameters

At 64 weeks, serum samples were analyzed for total cholesterol, triglyceride, high-density lipoprotein cholesterol, glutamic oxaloacetic transaminase, glutamic pyruvic transaminase, albumin, total protein, and uric acid using an automated blood chemistry analyzer (Film DRI CHEM 7000i; Fuji Film).

### Determinations of corticosterone in yolk samples

Eggs collected at 4-week intervals from 36 to 64 weeks were used to quantify yolk corticosterone levels as described elsewhere [27]. In brief, the separated yolks were pooled (3 yolks/replicate) were mixed with ethanol and the mixture was extracted at 37°C for 1 h in the incubator and then centrifuged. The supernatants were analyzed using a commercially available CORT ELISA kit (ADI-901-097; Enzo Life Science) per the manufacturer’s recommendation.

### Statistical analysis

Data were analyzed using the GLM procedure of the SAS (SAS Institute). The model included dietary treatment as a fixed variable. The least square means were calculated for each treatment. Due to the lack of differences among four treatments, yolk corticosterone levels measured at 36, 40, 44, 48, 52, 56, 60, and 64 weeks were combined for statistical analysis. Preplanned polynomial contrasts were employed to determine linear and quadratic effects of dietary protein levels. An alpha level of 0.05 was used to determine statistical significance.

## RESULTS

Final body weight of laying hens at 64 weeks of age was lowered (quadratic effect, p = 0.049) with decreasing protein levels (Table 1). However, body weight change and feed intake were not affected by dietary protein levels. Egg weight linearly declined with decreasing protein levels (p<0.001). Lowering dietary protein levels decreased (linear and quadratic effects, p<0.05) egg production and egg mass, but increased (linear and quadratic effects, p<0.05) feed conversion ratio. The percentages of soft and broken eggs tended to be reduced (quadratic effect, p = 0.055) as dietary protein level lowered, the values being higher in laying hens fed diets containing high and very low protein levels.

Decreasing dietary protein levels increased (quadratic effect, p = 0.033) eggs with thin eggshells at 44 weeks, but improved (linear effect, p<0.001) eggshell thickness at 52 weeks (Table 2). Eggshell strength was unaffected by dietary CP levels. Eggshell color was increased (linear effect, p = 0.015) at 52 weeks as dietary protein levels decreased. Lowering dietary protein levels increased Haugh unit, but decreased yolk color at all ages.

Relative weights of abdominal fat and internal organs (i.e., kidney, spleen, small intestine, and bursa of Fabricius) were not affected by dietary protein levels (Table 3). Decreasing dietary protein levels increased (quadratic effect, p = 0.059) liver weight but lowered the relative weights of ovary (quadratic effect, p = 0.037) and oviduct (linear effect, p = 0.047). Although dietary protein levels affected reproductive organs, number of large yellow follicles was not altered. Tibia characteristics (i.e., length, width, and breaking strength) in laying hens at 64 weeks were not affected by dietary protein levels.

Lowering dietary protein levels did not affect ileal villus height, but decreased (linear effect, p = 0.045) ileal crypt depth, leading to increased villus height:crypt depth (linear effect, p = 0.093; Table 4).

Decreasing dietary protein levels linearly increased the digestibility of dry matter at 45 and 55 weeks (p<0.05), and that of CP at 45 (p<0.001) and 55 (p = 0.053) weeks (Table 5). Laying hens fed diets containing lowered dietary protein levels exhibited decreased digestibility of ether extract and crude ash at 45 weeks (linear effect, p<0.05), but increased digestibility of ether extract at 64 weeks (linear effect, p = 0.068) and crude ash at 55 weeks (linear effect, p = 0.018).

Lowering dietary protein levels increased the concentrations of high-density lipoprotein cholesterol (linear and quadratic effects, p<0.05), but lowered (linear effect, p = 0.021) that of uric acid in serum samples at 64 weeks (Table 6). The concentrations of total cholesterol, triglyceride, glutamic oxaloacetatic transaminase, glutamic pyruvic transaminase, and total protein in serum samples were not altered by dietary protein levels.

Fresh excreta weight ranged from 83.75 to 95.03 g/d/hen at 45 weeks, from 72.20 to 82.24 g/d/hen at 55 weeks, and from 73.86 to 83.90 g/d/hen at 64 weeks (Table 7). Lowering dietary protein levels decreased (linear effect, p<0.001) nitrogen consumed, excreted, and retained at all ages. Linear regression analysis clearly showed the reduction of nitrogen excretion (Y) by each 2% reduction in dietary protein levels (x) at 45 weeks (Y = −0.242x+2.21, r^2^ = 0.988), 55 weeks (Y = −0.192x+ 1.61, r^2^ = 0.997), and 64 weeks (Y = −0.131x+1.30, r^2^ = 0.988). Odor gas emissions (i.e., carbon dioxide, ammonia, hydrogen sulfide, and trimethylamine) were not affected by dietary protein levels (Table 8). However, decreasing dietary protein levels linearly increased total volatile fatty acids in fecal digesta and this effect was only noted at 55 weeks (linear effect, p = 0.003).

**Figure 1:**
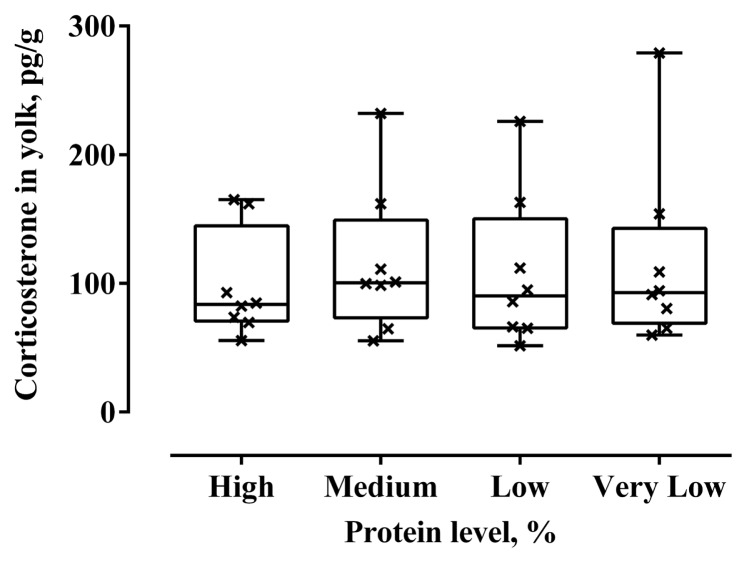
Effect of dietary protein levels on yolk corticosterone in laying hens. Data was obtained by pooling yolk corticosterone quantified in eggs collected at 4-week intervals from 33 to 64 week. Each box plot indicates the interquartile range with the median represented by the horizontal line within the box, while whiskers extend to show the full range of the data (represented by symbol X).

To determine whether dietary protein levels could affect stress responses in laying hens, we analyzed yolk corticosterone at 4-week intervals from 33 to 64 weeks. No differences in yolk corticosterone by dietary protein levels were found at all ages. Thus, all analyzed values for yolk corticosterone were pooled and presented (Figure 1).

## DISCUSSION

The present study aimed to investigate the long-term feeding effect of low-protein diets on laying performance, egg quality, nitrogen balance, odor emission, and yolk corticosterone in laying hens. Our results demonstrated that although body weight change was unaffected by dietary protein levels, laying hens performed less coupled with decreased reproductive organs with lowering dietary protein levels. However, low-protein diets improved ileal morphology, an indicator of gut health, and apparent total tract nutrient digestibility (e.g., dry matter or CP). Finally, low-protein diets did not affect yolk corticosterone but lowered nitrogen excretion. Taken together, these data suggest that low-protein diets fortified with limiting amino acids can be considered as an effective option to mitigate nitrogen emission during the laying cycle.

The findings of this study indicate that lowering dietary protein levels did not negatively affect body weight of laying hens, which is consistent with previous studies [11,28,29]. This can be attributed to the fact that all experimental diets were supplemented with limiting amino acids including lysine, methionine, and threonine which could have prevented the negative effect of low-protein diets, if any, on body weight. In addition, the observation that feed intake was not altered by dietary treatments indicates that nitrogen levels in low-protein diets might be sufficient to meet the requirements of laying hens for maintaining body composition or weight. However, lowering dietary protein levels decreased laying performance including egg weight, egg production, egg mass, and feed conversion ratio. This finding corroborates earlier studies showing that lowering dietary protein levels decreased laying performance including egg weight and production [30]. It is generally understood that nutritional factors including protein or energy levels could affect laying performance [31,32]. Thus, although all experimental diets had equal concentrations of limiting amino acids (i.e., lysine, methionine, threonine, and valine) and metabolizable energy, it is likely that suboptimal levels of nitrogen required for maintaining optimal laying performance could have led to decreased laying performance. In addition, impaired feed efficiency by dietary protein levels noted in this study indicates that feed consumed, especially diets with low and very low protein levels, was not efficiently utilized to lay eggs due to the lack of nitrogen for egg production. Our findings align with earlier studies [33,34] that low-protein diets decreased egg production and egg mass but increased feed conversion ratio in laying hens. In addition, our finding that lowering dietary protein levels lowered the relative weights of productive organs (i.e., ovary and oviduct) could also support the reduction in laying performance (i.e., egg weight, egg production, and egg mass).

Egg quality plays a crucial role in the commercial egg industry [35]. It is clear from this study that lowering dietary protein levels did not impair eggshell qualities. Instead, it is tempting to conclude that low-protein diets improved Haugh unit, an indicator of freshness, without impairing eggshell qualities. It is documented that eggshell quality is influenced by genetics, age, environment, and nutrition including calcium, phosphorus, vitamin D, trace minerals, and amino acids [35]. Thus, our study indicates that all nutrients (e.g., calcium, amino acids) needed to synthesize eggshell and albumen are adequately provided in laying hens fed suboptimal low-protein diets. In line with our findings, low-protein diets did not affect eggshell qualities in laying hens [11]. Of interest, lowering dietary protein levels produced pale brown eggs at 52 weeks of age. It is known that endogenously synthesized pigments (i.e., biliverdin and protoporphyrin) are deposited into eggshell at the final stage of egg formation [36]. Thus, it might be likely that low protein levels may affect pigment deposition process via reproductive organs rather than inhibiting the process of pigment synthesis. However, it should be kept in mind that eggshell color values in this study are all within the acceptable range [37]. Finally, we found that pale egg yolks were consistently produced in low-protein diet-fed laying hens. This is secondary to the concomitant decrease of corn gluten meals, rich in xanthophyll [38], in low-protein diets.

Gut morphology is considered an indicator of gut health in laying hens [39]. In this study, lowering dietary protein levels lowered crypt depth but tended to increase villus height:crypt depth in laying hens, indicating improved gut health. It has been documented that an increase in undigested proteins flowing into the distal intestine may cause the increase of harmful bacteria leading to impaired gut health [8,40]. Thus, it is likely that low protein levels in the diets of laying hens can lower undigested proteins in the distal intestine favoring a beneficial environment for gut microbiota.

We attempted to investigate whether dietary protein levels could affect apparent total tract digestibility of nutrients in laying hens. It is noted that the digestibility of dry matter and CP increased with decreasing dietary protein levels. This may be attributed to the enhanced digestibility of crystalline amino acids which were increased with decreasing dietary protein levels. In line with our findings, lowering dietary protein levels fortified with limiting amino acids improved the digestibility of protein and amino acids in laying hens [12,15,23]. In this study, we found that the digestibility of ether extracts and crude ash exhibited similar patterns. Lowering dietary protein levels decreased the digestibility of ether extracts and crude ash at 45 weeks but increased the digestibility at 55 or 64 weeks. It is known that fatty acids can form insoluble soaps with calcium rendering unavailable to the host, thus leading to reductions in the digestibility of ether extracts and calcium [41]. However, we noted that eggshell thickness and strength, as influenced by calcium availability, were not altered by dietary protein levels. Thus, a clear explanation on the contradictory results on the digestibility of ether extracts and crude ash at different ages is not readily available that needs to be addressed.

Serum parameters can reflect physiological and metabolic changes and are used as indicators for the nutritional status in chickens [42]. In this study, lowering dietary protein levels increased high-density lipoprotein cholesterol but decreased uric acid in serum samples. It is well documented that high-density lipoprotein cholesterol has been considered as beneficial cholesterol [43], indicating the health-promoting role of low-protein diets in cholesterol metabolism. Uric acid levels directly reflect protein and amino acid metabolism, and it is the main end product of nitrogen metabolism in poultry [44]. Therefore, the observed decrease in uric acid levels by low-protein diets noted in this study can be explained as enhanced nitrogen efficiency with low nitrogen intake. Our results align with an earlier study [45] showing that serum uric acid levels (from 3.02 mg/dL to 1.68 mg/dL) and nitrogen intake (from 2.93 g/bird/day to 2.23 g/bird/day) were declined with lowering dietary protein levels (from 17% to 14%) in laying hen. The effect of low-protein diets on serum uric acid levels was also reported elsewhere [46,47].

Nitrogen excretion influences environmental pollution and represents the efficiency of diet-origin protein utilization [1,3]. As expected, low-CP diets lowered nitrogen intake and nitrogen excretion, leading to a decrease in nitrogen retention. Our study corroborates with earlier studies in laying hens [23] and broiler chickens [48], suggesting that lowering protein levels in the diets of laying hens is the best nutritional strategy to lower nitrogen excretion. Indeed, linear regression analysis indicates that each 2% protein reduction in the diets of laying hens led to lowering the excreted nitrogen by 0.24 g/d/hen at 45 weeks, 0.19 g/d/hen at 55 weeks, and 0.13 g/d/hen at 64 weeks.

Odors and volatile fatty acids in fecal droppings are produced by the incomplete anaerobic digestion of nutrients like carbohydrates or proteins in manure [49,50]. Although low-protein diets clearly lowered nitrogen excretion in this study, they did not affect odor emissions (i.e., carbon dioxide, ammonia, H_2_S, and trimethylamine) but increased total volatile fatty acid production in fecal droppings. Low protein-mediated increase in total volatile fatty acids was only noted at 55 weeks of age. Our finding agrees with a study by Heo et al [23] who found that lowering dietary protein levels did not affect odor emissions in 33-week-old laying hens but disagrees with earlier studies those [14,15] which reported that odor emissions were lowered in laying hens fed low-protein diets. Our study differs from the earlier study [23] showing that low-protein diets did not affect total volatile fatty acids in pullets and laying hens. It is thus tempting to conclude that decreasing dietary protein levels may shift the compositions of distal gut microflora favoring the production of total volatile fatty acids on undigested nutrients in distal intestine.

Corticosterone is secreted in responses of hens to nutritional stressors such as dietary phosphorus levels, feed restriction and feed density [22,51,52]. Thus, we quantified yolk corticosterone as a noninvasive biomarker for assessing stress levels in laying hens. In this study, the concentration of yolk corticosterone was not affected by dietary protein levels. However, Heo et al [23] reported that low-protein diets-fed laying hens at 22 weeks of age, but not at 26 and 32 weeks of age, had decreased concentrations of yolk corticosterone. This suggests that low protein *per se* may not act as a nutritional stressor in laying hens. On the other hand, Park et al [22] showed that laying hens fed low *vs*. adequate phosphorus diets laid eggs with elevated yolk corticosterone. Thus, it is likely that stress responses of laying hens to low-nutrition diets are dependent on the nutrients.

## CONCLUSION

In conclusion, low-protein diets lowered laying performance and reproductive organs but did not affect eggshell qualities. Reducing dietary protein levels improved the digestibility of nutrients including CP and effectively decreased nitrogen excretion. Lowering dietary protein levels did not affect odor emissions but increased concentrations of total volatile fatty acids. Our study shows that lowering dietary protein levels fortified with limiting amino acids is the best nutritional approaches to mitigate nitrogen excretion. It is, however, needed to find nutritional solutions (e.g., exogenous enzymes) to lower odor emissions and to improve laying performance in laying hens fed low-protein diets.

## Figures and Tables

**Table 1:** Effect of dietary protein levels on laying performance in laying hens at 33 to 64 wk

Items	Protein levels (%)^1)^				SEM	p-value	
	
	High	Medium	Low	Very low		Linear	Quadratic
Initial BW (kg/hen)	1.92	1.97	1.97	2.01	0.05	0.268	0.976
Final BW (kg/hen)	1.88	1.97	1.96	1.85	0.05	0.647	0.049
BW change (kg/hen)	−0.04	0.01	−0.01	−0.16	0.07	0.251	0.155
ADFI (g/hen/d)	121.75	121.71	121.76	121.68	0.18	0.848	0.921
Egg weight (g)	62.98	62.92	61.93	60.88	0.40	<0.001	0.224
Egg production (%)	91.06	92.16	90.56	84.33	1.03	<0.001	0.001
Egg mass (g/d)	56.10	57.79	53.67	48.69	1.12	<0.001	0.004
FCR (g/g)	2.21	2.11	2.30	2.56	0.06	<0.001	0.003
Soft and broken egg (%)	0.311	0.192	0.226	0.486	0.096	0.200	0.055

1) High protein diet containing 18.5%, 18.0%, 17.0% in feeding phases I, II, and III; medium protein diet containing 16.5%, 16.0%, 15.0% in feeding phases I, II, and III; low-protein diets 14.5%, 14.0%, 13.0% in feeding phases I, II, and III; very low-protein diet containing 12.5%, 12.0%, 11.0% in feeding phases I, II, and III.

SEM, standard error of the mean; BW, body weight; ADFI, average daily feed intake; FCR, feed conversion ratio.

**Table 2:** Effects of dietary protein levels on egg quality in laying hens

Items	Protein levels (%)^1)^				SEM	p-value	
	
	High	Medium	Low	Very low		Linear	Quadratic
Eggshell thickness (mm)							
44 wk	0.41	0.39	0.40	0.40	0.01	0.876	0.033
52 wk	0.37	0.38	0.39	0.41	0.01	<0.001	0.452
64 wk	0.39	0.38	0.38	0.39	0.01	0.957	0.478
Eggshell strength (kgf)							
44 wk	5.52	5.46	5.47	5.60	0.11	0.612	0.418
52 wk	4.86	4.76	4.58	4.87	0.13	0.433	0.674
64 wk	4.30	4.09	4.33	4.65	0.18	0.118	0.145
Eggshell color (unit)							
44 wk	25.3	24.8	24.8	25.3	0.8	0.965	0.496
52 wk	22.2	24.4	25.1	25.0	0.8	0.015	0.179
64 wk	23.8	24.1	24.0	23.5	0.7	0.707	0.558
Haugh unit							
44 wk	93.7	92.9	94.1	95.3	0.7	0.092	0.228
52 wk	86.3	87.7	87.0	90.2	1.2	0.044	0.459
64 wk	83.2	84.2	84.4	86.3	0.9	0.024	0.625
Yolk color							
44 wk	8.63	8.58	8.31	8.42	0.09	0.028	0.364
52 wk	8.59	8.24	7.42	6.96	0.09	<0.001	0.573
64 wk	7.44	7.08	6.08	5.62	0.13	<0.001	0.686

1) High protein diet containing 18.5%, 18.0%, 17.0% in feeding phases I, II, and III; medium protein diet containing 16.5%, 16.0%, 15.0% in feeding phases I, II, and III; low-protein diets 14.5%, 14.0%, 13.0% in feeding phases I, II, and III; very low-protein diets containing 12.5%, 12.0%, 11.0% in feeding phases I, II, and III.

SEM, standard error of the mean.

**Table 3:** Effect of dietary protein levels on relative organ weight (g/100 g of BW) and tibia characteristics in laying hens (64 wk)

Items	Protein levels (%)^1)^				SEM	p-value	
	
	High	Medium	Low	Very Low		Linear	Quadratic
Organ weight							
Abdominal fat	2.91	3.08	3.64	3.81	0.44	0.112	0.998
Liver	1.79	2.00	2.13	1.95	0.10	0.185	0.059
Kidney	0.8	0.76	0.78	0.78	0.02	0.795	0.402
Spleen	0.11	0.12	0.10	0.10	0.02	0.461	0.653
Duodenum	0.37	0.34	0.42	0.41	0.03	0.179	0.807
Jejunum	0.48	0.50	0.55	0.51	0.05	0.493	0.585
Ileum	0.43	0.42	0.46	0.36	0.06	0.486	0.466
Bursa of Fabricius	0.08	0.07	0.05	0.06	0.03	0.641	0.762
Ovary	0.32	0.26	0.24	0.30	0.03	0.412	0.037
Oviduct	3.71	3.52	3.23	3.16	0.21	0.047	0.776
Number of large of yellow follicles	4.71	4.57	4.71	4.00	0.27	0.115	0.307
Tibia characteristics							
Fresh weight	0.71	0.67	0.64	0.67	0.02	0.196	0.150
Length (cm)	12.19	12.10	12.26	12.23	0.12	0.907	0.998
Width (cm)	0.76	0.75	0.74	0.73	0.01	0.213	0.948
Strength (kgf)	14.5	16.8	13.2	16.8	1.2	0.548	0.566

1) High protein diet containing 18.5%, 18.0%, 17.0% in feeding phases I, II, and III; medium protein diet containing 16.5%, 16.0%, 15.0% in feeding phases I, II, and III; low-protein diets 14.5%, 14.0%, 13.0% in feeding phases I, II, and III; very low-protein diets containing 12.5%, 12.0%, 11.0% in feeding phases I, II, and III.

SEM, standard error of the mean.

**Table 4:** Effect of dietary protein levels on ileal morphology in laying hens (64 wk)

Items	Protein levels (%)^1)^				SEM	p-value	
	
	High	Medium	Low	Very low		Linear	Quadratic
Villus height (VH) (μm)	865	876	902	879	25	0.552	0.495
Crypt depth (CD) (μm)	89.1	89.1	85.2	85.3	1.6	0.045	0.945
VH:CD	9.72	9.86	10.6	10.3	0.3	0.093	0.495

1) High protein diet containing 18.5%, 18.0%, 17.0% in feeding phases I, II, and III; medium protein diet containing 16.5%, 16.0%, 15.0% in feeding phases I, II, and III; low-protein diets 14.5%, 14.0%, 13.0% in feeding phases I, II, and III; very low-protein diets containing 12.5%, 12.0%, 11.0% in feeding phases I, II, and III.

SEM, standard error of the mean.

**Table 5:** Effects of dietary protein levels on apparent total tract digestibility in laying hens

Items	Protein levels (%)^1)^				SEM	p-value	
	
	High	Medium	Low	Very low		Linear	Quadratic
Dry matter (%)							
45 wk	71.1	71.2	73.6	73.1	0.7	0.016	0.658
55 wk	73.3	74.5	74.8	77.6	0.7	<0.001	0.247
64 wk	75.2	76.3	76.9	77.7	1.7	0.357	0.937
Crude protein (%)							
45 wk	32.9	35.6	41.1	42.1	1.7	<0.001	0.686
55 wk	45.8	44.5	46.9	50.3	0.7	0.053	0.192
64 wk	50.5	52.3	53.5	54.4	3.6	0.483	0.904
Ether extract (%)							
45 wk	89.8	89.4	89.6	85.6	0.9	0.008	0.085
55 wk	87.3	87.0	85.6	87.1	1.0	0.667	0.366
64 wk	87.6	88.0	88.0	93.5	1.2	0.068	0.013
Crude ash (%)							
45 wk	32.2	29.4	27.3	21.4	2.0	0.001	0.431
55 wk	37.4	41.8	40.8	46.7	2.4	0.018	0.744
64 wk	44.2	45.9	50.3	51.5	5.1	0.315	0.961

1) High protein diet containing 18.5%, 18.0%, 17.0% in feeding phases I, II, and III; medium protein diet containing 16.5%, 16.0%, 15.0% in feeding phases I, II, and III; low-protein diets 14.5%, 14.0%, 13.0% in feeding phases I, II, and III; very low-protein diets containing 12.5%, 12.0%, 11.0% in feeding phases I, II, and III.

SEM, standard error of the mean.

**Table 6:** Effect of dietary protein levels on serum parameters in laying hens (64 wk)

Items	Protein levels (%)^1)^				SEM	p-value	
	
	High	Medium	Low	Very low		Linear	Quadratic
TCHO (mg/dL)	63.1	60.4	65.0	68.0	5.8	0.475	0.655
HDLC (mg/dL)	18.6	16.3	19.6	23.4	1.5	0.012	0.047
TG (mg/dL)	422	343	357	307	78	0.369	0.109
GOT (U/L)	203	356	226	144	64	0.296	0.797
GPT (U/L)	29.7	28.7	26.9	26.7	1.6	0.137	0.963
Total protein (g/dL)	4.07	3.90	3.73	3.63	0.26	0.209	0.891
Uric acid (mg/dL)	2.61	2.56	1.96	1.10	0.47	0.021	0.398

1) High protein diet containing 18.5%, 18.0%, 17.0% in feeding phases I, II, and III; medium protein diet containing 16.5%, 16.0%, 15.0% in feeding phases I, II, and III; low-protein diets 14.5%, 14.0%, 13.0% in feeding phases I, II, and III; very low-protein diets containing 12.5%, 12.0%, 11.0% in feeding phases I, II, and III.

SEM, standard error of the mean; TCHO, total cholesterol; HDLC, high-density lipoprotein cholesterol; TG, triglycerides; GOT, glutamic oxaloacetic transaminase; GPT, glutamic pyruvic transaminase.

**Table 7:** Effects of dietary protein levels on nitrogen (N) balance (expressed as g/hen/d) in laying hens

Items	Protein levels (%)^1)^				SEM	p-value	
	
	High	Medium	Low	Very low		Linear	Quadratic
Fresh excreta							
45 wk	83.91	83.75	95.03	85.63	5.83	0.534	0.436
55 wk	77.99	72.20	76.45	82.24	4.67	0.424	0.227
64 wk	79.65	73.86	78.11	83.90	4.67	0.424	0.227
Nitrogen intake							
45 wk	2.695	2.412	2.071	1.810	0.010	<0.001	0.149
55 wk	2.589	2.180	1.954	1.679	0.057	<0.001	0.246
64 wk	2.449	2.138	1.931	1.563	0.054	<0.001	0.604
Nitrogen excretion							
45 wk	1.977	1.727	1.432	1.268	0.060	<0.001	0.441
55 wk	1.405	1.241	1.038	0.832	0.049	<0.001	0.677
64 wk	1.189	1.020	0.898	0.792	0.077	0.001	0.681
Nitrogen retention							
45 wk	0.718	0.685	0.639	0.541	0.060	0.037	0.590
55 wk	1.184	0.939	0.916	0.848	0.049	<0.001	0.081
64 wk	1.259	1.118	1.033	0.770	0.087	0.001	0.492

1) High protein diet containing 18.5%, 18.0%, 17.0% in feeding phases I, II, and III; medium protein diet containing 16.5%, 16.0%, 15.0% in feeding phases I, II, and III; low-protein diets 14.5%, 14.0%, 13.0% in feeding phases I, II, and III; very low-protein diets containing 12.5%, 12.0%, 11.0% in feeding phases I, II, and III.

SEM, standard error of the mean.

**Table 8:** Effects of dietary protein levels on odor emission in laying hens

Items	Protein levels (%)^1)^				SEM	p-value	
	
	High	Medium	Low	Very low		Linear	Quadratic
Carbon dioxide (ppm)							
45 wk	266	289	318	289	18	0.251	0.177
55 wk	686	501	544	527	66	0.158	0.218
64 wk	850	720	700	785	69	0.499	0.145
Ammonia (ppm)							
45 wk	3.20	3.00	2.67	2.00	0.53	0.155	0.717
55 wk	8.75	7.00	8.20	5.50	2.93	0.639	0.901
64 wk	11.6	12.0	10.0	4.00	4.30	0.299	0.570
Hydrogen sulfide (ppm)							
45 wk	0.15	0.20	0.22	0.16	0.04	0.670	0.355
55 wk	0.68	1.33	1.00	0.76	0.28	0.958	0.163
64 wk	1.30	2.04	1.30	1.80	0.42	0.688	0.776
Trimethylamine (ppm)							
45 wk	0.70	0.50	0.65	0.20	0.16	0.134	0.541
55 wk	9.80	6.25	9.00	5.67	2.73	0.502	0.972
64 wk	10.00	7.67	8.00	8.00	0.84	0.235	0.264
Total VFA (μM)							
45 wk	18.08	18.55	19.55	20.48	1.10	0.107	0.837
55 wk	10.85	14.37	17.56	20.62	2.23	0.003	0.920
64 wk	18.10	16.33	19.66	17.66	2.59	0.864	0.963

1) High protein diet containing 18.5%, 18.0%, 17.0% in feeding phases I, II, and III; medium protein diet containing 16.5%, 16.0%, 15.0% in feeding phases I, II, and III; low-protein diets 14.5%, 14.0%, 13.0% in feeding phases I, II, and III; very low-protein diet containing 12.5%, 12.0%, 11.0% in feeding phases I, II, and III.

SEM, standard error of the mean.

## Data Availability

Upon request, the datasets of this study can be available from the corresponding author.

## SUPPLEMENTARY MATERIAL

**Supplement 1**. Ingredients and calculated chemical composition of experimental diets fed to laying hens from 33 to 64 weeks of age.
