# Supplementary material for: Nitrogen balance and yolk corticosterone levels of laying hens fed low-protein diets from 33 to 64 weeks of age
Source: Anim Biosci. 2025 Dec 18;39(5):250814. doi: 10.5713/ab.250814 (PMC13175073; doi:10.5713/ab.250814)
Supplement: Supplementary file 1 [file ab-250814-Supplement-1.pdf]

**Supplement 1. Ingredients and calculated chemical composition of experimental diets fed to laying hens from 33 to 64 weeks of age.**

| | Phase 1 (33–45 wk) | | | | Phase II (46–55 wk) | | | | Phase III (56–64 wk) | | | |
|---|---|---|---|---|---|---|---|---|---|---|---|---|
| Treatment[1] | H | M | L | VL | H | M | L | VL | H | M | L | VL |
| Ingredient composition, % | | | | | | | | | | | | |
| Corn | 54.26 | 59.48 | 64.68 | 69.88 | 56.47 | 61.04 | 65.60 | 70.16 | 56.42 | 60.69 | 64.94 | 69.20 |
| Soybean meal | 19.71 | 16.16 | 13.55 | 9.88 | 20.90 | 17.22 | 13.55 | 9.88 | 21.37 | 17.63 | 13.90 | 10.17 |
| Tallow | 4.04 | 3.57 | 2.33 | 2.00 | 3.00 | 2.67 | 2.33 | 2.00 | 3.00 | 2.69 | 2.38 | 2.07 |
| Corn gluten meal | 8.00 | 6.33 | 2.33 | 1.00 | 6.40 | 4.60 | 2.80 | 1.00 | 5.14 | 3.42 | 1.71 | - |
| Limestone | 11.41 | 11.44 | 11.47 | 11.50 | 10.62 | 10.65 | 10.67 | 10.70 | 11.50 | 11.53 | 11.55 | 11.58 |
| Monocalcium phosphate | 1.63 | 1.64 | 1.64 | 1.65 | 1.80 | 1.82 | 1.83 | 1.85 | 1.80 | 1.82 | 1.83 | 1.85 |
| Sodium bicarbonate | - | - | - | - | 0.18 | 0.19 | 0.19 | 0.20 | 0.22 | 0.21 | 0.21 | 0.20 |
| Sodium chloride | 0.40 | 0.40 | 0.40 | 0.40 | 0.25 | 0.24 | 0.23 | 0.22 | 0.25 | 0.27 | 0.27 | 0.30 |
| Choline chloride | 0.05 | 0.05 | 0.05 | 0.05 | 0.05 | 0.05 | 0.05 | 0.05 | 0.05 | 0.05 | 0.05 | 0.05 |
| Vitamin premix[2] | 0.10 | 0.10 | 0.10 | 0.10 | 0.10 | 0.10 | 0.10 | 0.10 | 0.10 | 0.10 | 0.10 | 0.10 |
| Mineral premix[3] | 0.10 | 0.10 | 0.10 | 0.10 | 0.10 | 0.10 | 0.10 | 0.10 | 0.10 | 0.10 | 0.10 | 0.10 |
| L-lysine, HCL 56% | 0.30 | 0.49 | 0.69 | 0.88 | 0.30 | 0.23 | 0.42 | 0.62 | - | 0.20 | 0.41 | 0.61 |
| DL-Methionine, 99% | - | 0.04 | 0.09 | 0.13 | 0.10 | 0.15 | 0.19 | 0.24 | 0.05 | 0.13 | 0.20 | 0.28 |
| L-Threonine, 99% | - | 0.08 | 0.17 | 0.25 | - | 0.08 | 0.17 | 0.25 | - | 0.09 | 0.17 | 0.26 |
| L-Valine, 99% | - | 0.11 | 0.22 | 0.33 | - | 0.11 | 0.22 | 0.33 | - | 0.11 | 0.21 | 0.32 |
| Chemical composition, % | | | | | | | | | | | | |
| Dry matter | 89.13 | 88.97 | 88.80 | 88.63 | 88.88 | 88.74 | 88.60 | 88.46 | 88.64 | 88.53 | 88.42 | 88.31 |
| AMEn[4], kcal/kg | 2800 | 2800 | 2800 | 2800 | 2,750 | 2,750 | 2,750 | 2,750 | 2,700 | 2,700 | 2,700 | 2,700 |
| Crude protein | 18.5 | 16.5 | 14.5 | 12.5 | 18.0 | 16.0 | 14.0 | 12.0 | 17.0 | 15.0 | 13.0 | 11.0 |
| Energy to protein ratio | 151 | 170 | 193 | 224 | 153 | 172 | 196 | 229 | 162 | 183 | 212 | 250 |
| Ether extract | 6.21 | 5.83 | 5.46 | 5.08 | 5.24 | 5.01 | 4.78 | 4.55 | 5.12 | 4.89 | 4.66 | 4.43 |
| Ash | 15.0 | 14.8 | 14.7 | 14.5 | 14.4 | 14.3 | 14.2 | 14.0 | 15.6 | 15.5 | 15.3 | 15.2 |
| Calcium | 4.40 | 4.40 | 4.40 | 4.40 | 4.20 | 4.20 | 4.20 | 4.20 | 4.40 | 4.40 | 4.40 | 4.40 |
| Total phosphorus | 0.72 | 0.71 | 0.69 | 0.68 | 0.75 | 0.75 | 0.75 | 0.75 | 0.77 | 0.75 | 0.74 | 0.72 |
| Available phosphorus | 0.50 | 0.50 | 0.50 | 0.50 | 0.40 | 0.40 | 0.40 | 0.40 | 0.40 | 0.40 | 0.40 | 0.40 |
| Lysine | 0.93 | 0.93 | 0.93 | 0.93 | 0.80 | 0.80 | 0.80 | 0.80 | 0.79 | 0.79 | 0.79 | 0.79 |
| Methionine + Cysteine | 0.64 | 0.64 | 0.64 | 0.64 | 0.73 | 0.73 | 0.73 | 0.73 | 0.64 | 0.64 | 0.64 | 0.64 |
| Threonine | 0.67 | 0.67 | 0.67 | 0.67 | 0.66 | 0.66 | 0.66 | 0.66 | 0.65 | 0.65 | 0.65 | 0.65 |
| Valine | 0.87 | 0.87 | 0.87 | 0.87 | 0.86 | 0.86 | 0.86 | 0.86 | 0.81 | 0.81 | 0.81 | 0.81 |

[1] H: High protein diet containing 18.5%, 18.0%, 17.0% in feeding phases I, II, and III; M: medium protein diet containing 16.5%, 16.0%, 15.0% in feeding phases I, II, and III; L: low protein diets 14.5%, 14.0%, 13.0% in feeding phases I, II, and III; VL: very low protein diet containing 12.5%, 12.0%, 11.0% in feeding phases I, II, and III.

[2] Vitamin and mineral premixes provided the following quantities per kg of complete diets: vitamin A, 36,000 IU; vitamin D3, 6,000 mg; vitamin E, 160 IU; vitamin K, 10 mg; vitamin B1, 10 mg; vitamin B 240 mg; vitamin B6, 16 mg; vitamin B12, 0.12 mg; biotin, 0.40 mg; pantothenic acid, 100 mg; niacin, 180 mg; Fe, 200 mg; Mn, 100 mg; Zn, 31.25 mg; Cu, 75 mg; I, 0.63 mg; and Se, 0.50 mg

[3)] Mineral premix provided the following nutrients per kg of diet: Fe as iron sulfate, 70 mg; Mn as manganese sulfate, 80 mg; Zn as zinc oxide, 60 mg; Cu as copper sulfate, 8 mg; Co as cobalt sulfate, 0.13 mg; I as calcium iodate, 1 mg; Se as selenium yeast, 0.20 mg.

[4)] AMEn, nitrogen-corrected apparent metabolizable energy, kcal/kg.
